# Dataset of the lab-scale 3-axis winding machine integrated with the portable real-time winding angle measurement system

**DOI:** 10.1016/j.dib.2022.108731

**Published:** 2022-11-08

**Authors:** Quanjin Ma, M.R.M. Rejab, M.S. Idris

**Affiliations:** a Structural Performance Materials Engineering (SUPERME) Focus Group, Faculty of Mechanical & Automotive Engineering Technology, Universiti Malaysia Pahang, 26600 Pekan, Pahang, Malaysia; b School of Mechanical Engineering, Ningxia University, 750021 Yinchuan, China

**Keywords:** Filament winding technique, Experimental assessment, Winding performance, CFRP, Filament-wound composite tube, Winding angle, Real-time winding angle measurement system, Technical specification

## Abstract

This article presents three datasets related to the laboratory scale 3-axis filament winding machine. The winding experimental tests are described on the range of winding angle, winding accuracy of programmed G-codes, and linear and rotation speeds in raw data. The real-time winding angle measurement system is developed to monitor and measure the winding angle of filament-wound carbon-fiber reinforced plastics (CFRP) tubes. Two winding patterns are provided as dry and wet winding processes. Moreover, an experimental test of a real-time winding angle measurement system is captured and analyzed. The *i*-winder app controls the winding machine through a Bluetooth module, which is programmed by MIT App Inventor. The data presented in this article can have a benchmark for developing a multi-axis filament winding machine. It is provided an inexpensive and open-source control system and is embedded in a real-time winding angle measurement system. The experimental assessment data can be found in this article [1]. The data is available in the cloud-based Mendeley Data repository [2].

**Specifications Table**

**Table:** 

Subject	Composites, fabrication method, filament winding
Specific subject area	Lab-scale 3-axis winding machine, dry and wet winding, winding angle, CFRP, filament wound composite tube
Type of data	Table Image Graph Figure
How the data were acquired	The lab-scale 3-axis filament winding machine is manufactured with an inexpensive control system. The hardware is mainly used the Arduino UNO microcontroller and CNC V1 shield module. The Universal G-code Sender software is obtained to send programming G-codes, which perform the dry and wet winding process with hoop and helical patterns. The real-time winding angle measurement system is embedded in the portable 3-axis winding machine. The hardware is mainly used Raspberry Pi 3B+ and Arducam 5MP OV5647 camera modules. The winding angle measurement system adopted the OpenCV software, which is programmed by Python. The i-winder app is developed to control the 3-axis winding machine using a Bluetooth module, which can perform the complete winding process.
Data format	Raw data Analyzed data
Description of data collection	• Four types of customized G-codes are programmed as the single winding path, which are ±30°, ±45°, ±60°, and ±75° winding angle G-codes, saved as corresponding text format. • The accuracy of the winding angle is studied on ±30°, ±45°, ±60°, and ±75° winding angles. It is measured by Dino-Lite Edge Digital Microscope and saved in word format. • The real-time winding angle measurement system is programmed using Python and saved in py format. • The real-time winding angle detection and measurement results were captured and analyzed. The winding angle data is measured and saved in PNG format.
Data source location	Institution: Faculty of Mechanical & Automotive Engineering Technology (FTKMA), Universiti Malaysia Pahang City/Town/Region: Pekan, Pahang Country: Malaysia Latitude and longitude (and GPS coordinates, if possible) for collected samples/data: Latitude: 3.7178 Longitude: 103.1193.
Data accessibility	The data is available in the Mendeley Data (https://data.mendeley.com/) Direct URL to data: https://data.mendeley.com/datasets/zyysdssxz2
Related research article	Quanjin, M., Rejab, M. R. M., Kumar, N. M., & Idris, M. S, Experimental assessment of the 3-axis filament winding machine performance, Results Eng. 2 (2019) 100017. https://doi.org/10.1016/j.rineng.2019.100017.

## Value of the Data

• Filament winding process is one of the traditional composite fabrication methods, which has increasingly been used in many application fields [3, 4]. The mechanical drawings of the portable 3-axis filament winding machine are considered as the reference case. The matched control system is developed, which has huge potential to reduce costs and break down software barriers [5].
• Technicians, R&D engineers, and researchers who work on filament winding process and filament wound composite products can benefit from these data.
• The portable real-time winding angle measurement system is firstly used to monitor and measure the winding angle, which is assembled on the laboratory scale winding machine. It is valuable to improve the precision of winding angles and develop filament winding equipment accessories.
• The *i*-winder app is developed to control the 3-axis winding machine using a Bluetooth module, which is programmed by MIT App Inventor. These data can be used in integrating the Internet of things (IoT) technology on the multi-axis winding machine [6]. It is valuable for understanding the technical feasibility research on winding equipment embedded with IoT technology.
• The real-time system experimental test is detected and measured on the captured image, which is used by the OpenCV software. These data can be used to develop a real-time detection system that integrates artificial intelligence (AI) computer vision technology.

## 1 Data Description

The file names are related to 1-The lab-scale 3-axis filament winding machine, 2-The real-time winding angle measurement device, and 3-The i-winder app, which are discussed as follows:

### 1.1 1-The lab-scale 3-axis filament winding machine

The file contains the mechanical drawings of the 3-axis filament winding machine, control system wiring diagram of the winding machine, technical specification, instruction operation manual, G-codes for the resin curing process, single loop winding G-codes with ±30°, ±45°, ±60°, ±55°and ±75° winding angle on tubular structure (txt. format), the UGS setting value (JPG. format), the wet winding process video (mov. format), and the G-code generator with different winding angles (excel. format).

### 1.2 2-The real-time winding angle measurement device

The file provides the mechanical drawings of the real-time winding angle measurement system, control system wiring diagram of the measurement device, measurement results, measurement system process video (mov. format), G-codes of linear movement (txt. format), winding angle line detection code (py. format).

### 1.3 3-The *i*-winder app

The file has the *i*-winder app installed in Android (apk. format), the app exported from MIT App Inventor (aia. format), *i*-winder app blocks, and two screen interface images of the *i*-winder app (PNG. format).

## 2 Experimental Design, Materials, and Methods

### 2.1 Materials and method

3K carbon fiber spool is supplied by Mitsubishi Rayon Co., Ltd, which offers a 3 mm width, 7 μm filament diameter, and 1.79 g/cm^3^ [1]. The resin system is used the EpoxAmite^TM^ 100 epoxy laminating system with 102 Medium Hardener, supplied by Smooth-on, which is mixed as the recommended weight ratio of 100:29. Dry and wet winding process is carried out, which is performed by the portable 3-axis winding machine. For the wet winding process, the mandrel is covered with three layers to provide easily demolding process: normal tissue layer, packing tape layer, and Teflon layer.

### 2.2 The lab-scale 3-axis filament winding machine

**Fig. 1:**
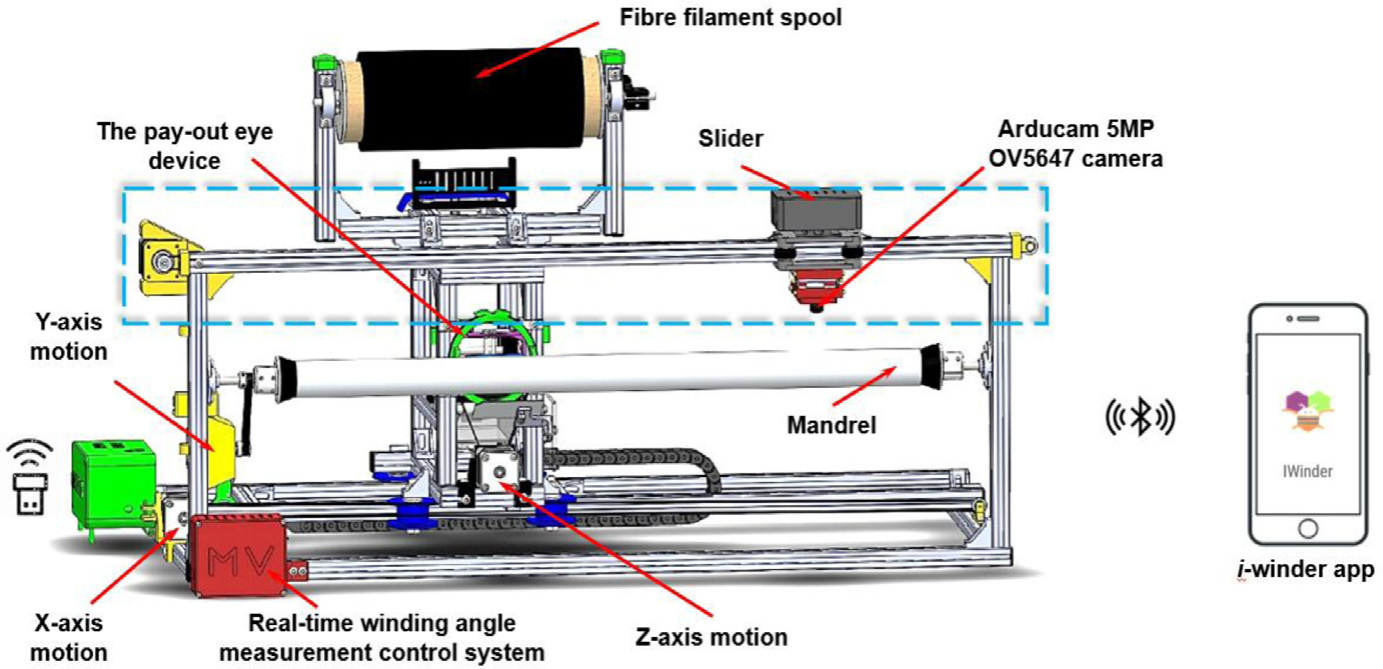
Schematic diagram of the lab-scale 3-axis filament winding machine integrated with the real-time winding angle measurement device

The portable 3-axis filament winding machine is developed and manufactured with an inexpensive, open-source control system. The hardware section is mainly used the Arduino UNO microcontroller and CNC V1 shield module. The software section is adopted the Universal G-code Sender software, which can send the programmed G-codes to perform the winding process. For the technical specification, the winding angle of the applied machine ranges from 20° to 85°, which offers 0.35-0.62° with a 2.25-8.68% standard deviation. Fig. 1 illustrates the 3-axis filament winding machine integrated with a real-time winding angle measurement device.

### 2.3 Experimental test of winding angle accuracy assessment

Four winding angles are used to evaluate winding performance, which are ±30°, ±45°, ±60°, and ±75° winding angles. The winding angle is measured using the protractor after the dry winding process, which is initially used to assess the winding performance. The winding angle of the filament wound tube is measured using the AM4815 Dino-lite edge microscope after fully cured from the wet winding process.

### 2.4 Experimental test of a real-time winding angle measurement device

The device mainly consists of hardware and software sections. The hardware section is adopted Raspberry Pi 3B+ and Arducam 5MP OV5647 camera module [7]. The software section is used OpenCV software. The dry winding process and real-time winding angle measurement process are proceeded simultaneously. The experimental results are obtained as captured images and collected data when two procedures are completed. The green color line is marked as the measurement winding angle within the allowable tolerance range. The red color line is represented the exceeding the allowable tolerance range. In addition, the actual measurement winding angle is collected in the software interface.

### 2.5 Development procedure of the *i*-winder app

The *i*-winder app is developed to perform the winding process through a Bluetooth module. The software project is created with two main screen viewers. The operator can install the *i*-winder app and open it when the Bluetooth module is successfully connected to the mobile phone. The operator inputs several critical parameters, such as thickness, length, bandwidth, winding angle, and z-axis rotation speed, which can send commands to perform the winding process. Two screen viewers are designed in the “Designer” part of the MIT App Inventor interface, and many blocks are assigned and programmed, such as parameter definition procedure, homing procedure, platform procedure, delay procedure, start procedure, Bluetooth connection procedure, manual operation procedure, and G-code procedure.

## Ethics Statements

This work has not involved any use of human subjects, animal experiments, and data collected from social media platforms.

## Data Availability

The data is hosted on Mendeley Data: https://data.mendeley.com/datasets/zyysdssxz2 (Version 2 data).

## CRediT Author Statement

**Quanjin Ma**: Original Draft, Writing – reviewing & editing, Conceptualization, Methodology, Software, Investigation, Validation; **M.R.M. Rejab**: Resources Conceptualization, Investigation, Supervision, Project administration, Funding acquisition; **M.S. Idris**: Software, Validation, Data curation.

## Declaration of Competing Interest

The authors declare that they have no known competing financial interests or personal relationships that could have appeared to influence the work reported in this paper.

## Data Availability

Dataset of laboratory scale 3-axis filament winding machine integrated with the real-time winding angle measurement system and i-winder app (Original data) (Mendeley Data)
